# WNT-β Catenin Signaling as a Potential Therapeutic Target for Neurodegenerative Diseases: Current Status and Future Perspective

**DOI:** 10.3390/diseases11030089

**Published:** 2023-06-25

**Authors:** Kakarla Ramakrishna, Lakshmi Vineela Nalla, Dumala Naresh, Kojja Venkateswarlu, Matte Kasi Viswanadh, Buchi N. Nalluri, Guntupalli Chakravarthy, Sajusha Duguluri, Payal Singh, Sachchida Nand Rai, Ashish Kumar, Veer Singh, Santosh Kumar Singh

**Affiliations:** 1 KL College of Pharmacy, Koneru Lakshmaiah Education Foundation Deemed to be University (KLU), Green Fields, Vaddeswaram, Guntur 522502, India; kakarlaramakrishna@kluniversity.in (K.R.); lakshmivineela@kluniversity.in (L.V.N.); nareshpharma2020@kluniversity.in (D.N.); mkasiviswanadh@kluniversity.in (M.K.V.); buchinalluri@kluniversity.in (B.N.N.); chakra_varthi123@kluniversity.in (G.C.); 2 Department of Pharmaceutical Engineering and Technology, Indian Institute of Technology, IIT BHU, Varanasi 221005, India; kvenkateswarlu.rs.phe19@iitbhu.ac.in; 3 Department of Biotechnology, Bharathi Institute of Higher Education and Research, Chennai 600073, India; sajushalina@gmail.com; 4 Department of Zoology, Mahila Maha Vidyalaya, Banaras Hindu University, Varanasi 221005, India; payalsingh200012@gmail.com; 5 Centre of Experimental Medicine and Surgery, Institute of Medical Sciences, Banaras Hindu University, Varanasi 221005, India; 6 ICMR-Rajendra Memorial Research Institute of Medical Sciences, Agamkuan, Patna 800007, India; ashish.rmrims@icmr.gov.in (A.K.); veersingh.rs.bce17@itbhu.ac.in (V.S.)

**Keywords:** WNT, β-catenin, homeostasis, neurogenesis, neurodegenerative diseases

## Abstract

Wnt/β-catenin (WβC) signaling pathway is an important signaling pathway for the maintenance of cellular homeostasis from the embryonic developmental stages to adulthood. The canonical pathway of WβC signaling is essential for neurogenesis, cell proliferation, and neurogenesis, whereas the noncanonical pathway (WNT/Ca^2+^ and WNT/PCP) is responsible for cell polarity, calcium maintenance, and cell migration. Abnormal regulation of WβC signaling is involved in the pathogenesis of several neurodegenerative diseases such as Alzheimer’s disease (AD), Parkinson’s disease (PD), Huntington’s disease (HD), amyotrophic lateral sclerosis (ALS), multiple sclerosis (MS), and spinal muscular atrophy (SMA). Hence, the alteration of WβC signaling is considered a potential therapeutic target for the treatment of neurodegenerative disease. In the present review, we have used the bibliographical information from PubMed, Google Scholar, and Scopus to address the current prospects of WβC signaling role in the abovementioned neurodegenerative diseases.

## 1. Introduction

The wingless-type mouse mammary tumor virus integration site (WNT)-βcatenin (WβC) signaling pathway is involved from embryonic development to adult-stage tissue regulation which is closely associated with multiple diseases, including neurodegenerative diseases, wound healing, bone diseases, hair loss, and chronic obstructive pulmonary disease, and many more cancers [1,2]. Neurodegenerative diseases such as Parkinson’s disease (PD), Alzheimer’s disease (AD), Huntington’s disease (HD), multiple sclerosis (MS), amyotrophic lateral sclerosis (ALS), and spinal muscular atrophy (SMA) incidences are globally increasing day by day. These neurological abnormalities are raised due to the degeneration of neurons. Several attempts were made to halt these pathological abnormalities. The plethora of investigations suggests that WβC signaling alterations contribute to the pathophysiology of these neurodegenerative diseases. Physiologically, WβC signaling plays a vital role in neuronal proliferation and neurogenesis [3]. Due to the neurogenesis property of WβC signaling, targeting this pathway rather than other pathological events could be a promising approach to prevent neurodegeneration and enhance neuronal proliferation and neurogenesis. Hence, in the present review, we have discussed the current understanding of WβC signaling in neurodegenerative diseases.

The wβC pathway is activated in two ways. The first one is a noncanonical pathway (WNT/Ca^2+^ and WNT/planar cell polarity (PCP)), where the WNT pathway works independently of β-catenin mediated activation of T-cell factor (TCF)/lymphoid enhance-binding factor (LEF) [4]. The second one is a canonical pathway, which is also termed as WNT/β-catenin pathway. This pathway works based on the activation of TCF/LEF transcription factors [5]. The canonical WNT pathway mainly controls cell proliferation, whereas the noncanonical WNT pathways regulate cell polarity and migration [6,7].

Structurally, the WβC system is comprised of four major segments, including extra cellular signal, membrane segment, cytoplasmic segment, and nuclear segment [6]. WNT proteins (WNT1, WNT3a, and WNT5a) are present on extra cellular signal and acts as a ligand to activate the WβC pathway [8]. The WNT receptors, such as low-density lipoprotein receptor-related protein (LRP) 5/6 (LRP 5/6) and Frizzled (FZD), are present in the cell membrane segment [9]. The cytosolic segment of WβChas multiple proteins, such as glycogen synthase kinase-3β (GSK-3β), casein kinase 1 (CK1), disheveled (Dvl), adenomatous polyposis coli (APC), and AXIN which facilitates the activation and translocation of β-catenin from the cytosol to nucleus thereby triggers WβC signaling [10]. The nuclear segment mainly includes β-catenin, TCF/LEF family members, and β-catenin downstream target genes, such as matrix metalloproteinases (MMPs) and c-Myc activation [6,10]. Endogenous and exogenous modulation (with agonists or antagonists) of any of these four segments could result in activation or inhibition of WβC signaling.

The noncanonical WβC signaling works as WβC independent pathway. There are two noncanonical pathways involved, one is WNT/PCP, and the second one is WNT/calcium pathway (Wnt/Ca^2+^ pathway) [11]. These two pathways work independently or work together to exhibit β-catenin-like functions but do not involve β-catenin. Unlike the canonical pathway, in the noncanonical pathway, WNT ligands do not require LRP5/6 as a coreceptor, but they use FZD as their receptor to activate or shutdown the noncanonical pathways [12]. Disheveled (Dsh) is activated as a result of WNT ligand interaction with FZD receptors. The Dishevelled-associated activator of morphogenesis 1 (Daam1) is activated by the active Dsh, and it subsequently activates the guanosine triphosphatases (GTPases) RhoA and Rac, as well as their respective downstream substrates Rho-associated kinase (ROCK) and janus kinase 1 (JNK1). Additionally, ROCK and JNK1 promote actin in the cytoskeleton which plays a crucial role in the cell proliferation [13]. In addition, JNK1 also activates c-jun in the nucleus, which participates in a variety of transcriptional activities. Collectively, activation of the Wnt/PCP pathway is responsible for the asymmetric distribution of cytoskeleton and cell polarization by maintaining cell planar polarity and cytoskeletal reorganization, respectively [14].

**Figure 1:**
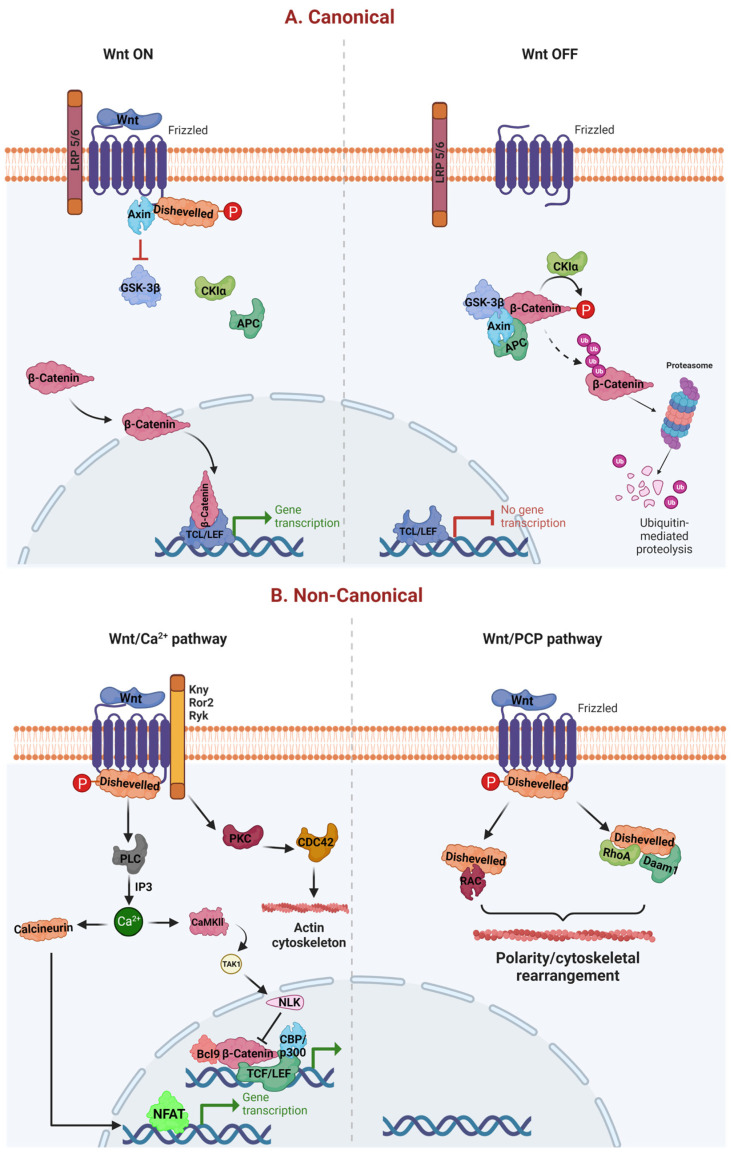
Canonical and noncanonical WβC signaling. (**A**) represents the canonical pathway of WβC signaling. WNT canonical pathway is activated when WNTs bind to FZD and LRP 5/6, leading to failure of destruction complex (Axin, CK1, APC, and GSK-3β), enabling the β-catenin translocation from cytoplasm to the nucleus [21]. Oppositely, the absence of WNT facilitates the activation of the destruction complex leading to the degradation of β-catenin, thereby preventing the β-catenin mediated transcription. (**B**) represents the noncanonical activation of WβC signaling. WNT/Ca^2+^ and WNT/PCP pathways are activated in the presence of WNT ligands. However, both these canonical pathways do not require β-catenin for their transcriptional activities [22].

WNT-dependent increases in Ca^2+^ levels are achieved either by inhibiting cyclic guanosine monophosphate (cGMP)-dependent protein kinase (PKG), which prevents Ca^2+^ release in unstimulated cells or by activating phospholipase C (PLC), which raises inositol 1,4,5-triphosphate (IP3) [15]. Intracellular Ca^2+^ further activates protein kinase C (PKC), calcium/calmodulin-dependent kinase II (CamKII), and calcineurin. CamKII promotes transform growth factor-β (TGF-β)-activated kinase (TAK1) and Nemo-like kinase (NEMO), which can phosphorylate and inactivate TCF and inhibit WβC signaling [16,17]. Further, calcineurin uncovers the nuclear localization of the nuclear factor of activated T cells (NFAT), allowing it to enter the nucleus and activate gene expression [18]. Eventually, NFAT activates the cyclic adenosine monophosphate (cAMP) response element-binding protein (CREB) mediated transcription process [19]—owing to multiple physiological and pathological roles, particularly cell proliferation, survival, and migration. The WNT signaling is critically regulated by WNT ligands. When WNTs are bound to FZD or LRP 5/6 on the cell membrane, it leads to recruitment of the destruction complex (APC, CK1, GSK-3β, and AXIN) to the cell membrane, fail to degrade the cytosolic β-catenin. Therefore, this cytosolic β-catenin translocates to the nucleus, where it interacts with TCF/LEF, thereby regulating the different functions [20,21]. In contrast to WNT on signaling, the absence of WNTs allows the FZD and LRP5/6 in their native place in WNT off signaling. This enables the destruction complex to degrade the β-catenin, which eventually prevents the translocation of β-catenin from the cytoplasm to the nucleus. Therefore, β-catenin is unable to interact with its nuclear TCF/LEF genes [4]. However, under physiological conditions, these WNT off and WNT on pathways are homeostatically regulated (Figure 1).

The prevalence rates of neurodegenerative diseases, such as AD, PD, SMA, HD, ALS, and MS, are rapidly growing [6,23,24,25]. WβC signaling is an essential component of neuronal proliferation and neurogenesis [3]. Therefore, understanding the diverse role of WβC signaling in the pathogenesis of neurodegenerative diseases is crucial to develop promising therapeutic interventions. Pathological events such as oxidative stress and inflammation cause neurodegeneration. These two events alone or combinedly cause neurodegeneration. Many of the phytochemicals, new chemical entities, and drugs have been evaluated against these events for their potential to ameliorate these neurodegenerative diseases. However, none of the compounds have been approved for the management of these neurodegenerative diseases in clinics. WβC signaling is known to inhibit oxidative stress and inflammation in neurons, thereby preventing neurodegeneration and concomitantly enhancing neuronal proliferation [5,26]. The Wnt/β-catenin axis is essential for the growth and homeostasis of the nervous system, and disruption of this axis has been linked to neurodegeneration. The majority of the neuronal activities that are compromised in these illnesses would be improved by the modulation of Wnt/β-catenin signaling in the brains of neurodegenerative diseases [27,28]. Therefore, the Wnt/β-catenin signaling pathway is an appealing target for neurodegenerative disease treatment since abnormal regulation of the pathway is closely linked to a variety of neurodegenerative diseases, including AD, PD, HD, SMA, MS, and ALS. Therefore, targeting this signaling for therapeutic purposes could offer both neuroprotection and neurorepair. In the present review, we have comprehensively summarized WβC signaling role in the pathogenesis or pharmacological agent against neurodegenerative diseases.

## 2. WβC Signaling in Alzheimer’s Disease

Alzheimer’s disease is accompanied by cognitive and memory deficits due to the accumulation of amyloid-β (Aβ) and tau-containing neurofibrillary tangles, oxidative stress, inflammation, mitochondrial dysfunction, and mutations on chromosomes such as APP (chromosome 21), presenilin 1 (chromosome 14), presenilin 2 (chromosome 1), and APOEe4 gene chromosome 19 result in the formation of Aβ plaques, by interfering with γ secretase [29,30,31,32,33,34,35,36]. Amyloid precursor protein (APP) cleaves the β-amyloid peptide by the action of α, β, γ secretase proteases [37]. APP cleavage is sequentially mediated by β-secretase and then by and γ cleavage results in the formation of β-amyloid containing 42 amino acids (Aβ42), which eventually forms β-amyloid aggregates at meninges, cerebral vessels, and gray matter [38]. The formation of neurofibrillary tau tangles is one of the pathological hallmarks of AD. Microtubules along the neuronal axons are crucial for intracellular transport. Tau protein stabilizes the axonal microtubules and promotes the extracellular aggregation of β-amyloid, which further hyperphosphorylates tau, thereby enhancing tau aggregation in the hippocampus [39]. It has been reported that AD doubles every 5 years after 65 years, and prevalence rates are raised to 10% at the age of 65 and 45% at the age of 50 [40]. AD symptoms vary from the stage of disease short-term memory loss, impairments in problem-solving, loss of execution, disorganization, lack of motivation, social withdrawal, psychosis, olfactory dysfunction, extrapyramidal motor problems, and sleep disturbances are accompanied by initial to later stages of disease progression [41]. The current treatment for AD is the administration of choline esterase inhibitors, n-methyl-D-asprtate (NMDA) antagonists, and choline esterase inhibitors such as donepezil, galantamine, and rivastigmine [42]. Apart from their beneficial roles, they also cause nausea, vomiting, diarrhea, increased vagal tone, syncope, bradycardia, and other cardiac problems may occur. Moreover, these agents only provide symptomatic relief [41,43]. To date, there are no disease-modifying agents to treat AD [44]. Therefore, neurorestorative therapies need to be studied in AD to prevent neurodegeneration and its associated complications [45,46].

A plethora of studies indicate that WβC signal impairment led neurons to become more susceptible to Aβ mediated apoptosis and stimulation of WβC free the Aβ induced toxicity in the brain in multiple ways [28,47,48,49]. Among them, synaptic damage enhances the loss of neuronal connections [50,51]. Early stages of AD are associated with synaptic loss and have a strong impact on learning and memory behavior [52]. Long-term potentiation (LTP) is required to build learning and memory behaviors. WNT proteins modulate neuronal transmission at pre and post-synapse by promoting LTP, thereby beginning synapse formation [53]. Endogenous WNT agonists such as WNT 1, WNT 2, WNT 3A, and WNT 7a/b increase the LTP [54], and WNT antagonists such as DKK1 and SFRP3 are reported to impede the LTP [55]. Aside from WNTs, LRP6 is a WNT co-receptor expressed on the excitatory synapses and responsible for excitatory synapse development and cognition [56]. Together, WNTS and LRP6 regulate synapse formation and function. AD is manifested by hippocampus-mediated memory loss, particularly novel object recognition memory (NORM) [57]. The latest evidence reports suggest that the activation of the WβC pathway enhances hippocampal neurogenesis [58]. DKK1, an inhibitor of WβC signaling, binds with LRP6 and blocks WβC signaling, resulting in impairment in hippocampus-mediated NORM by reducing β-catenin, c-myc, WNT 7, cyclin D, and PSD95 [59]. Studies to confirm the role of DKK1 in the inhibition of WβC signaling revealed that the induction of DKK1 expression causes memory impairment, synaptic dysfunction, and synaptic loss in the hippocampus [55]. Together, these findings confirm that LRP6 positively and DKK1 negatively regulate the WβC signaling mediated synaptic plasticity [59]. Therefore, these findings suggest that WβC signaling regulates the hippocampal-mediated NORM. Accumulation of amyloid plaques between neurons in the brain hampers neuronal communication. APP processing to amyloid plaques is mediated by β-site APP cleaving enzyme 1 (BACE 1). BACE1 enzyme, therefore, promotes the Aβ42 production and aggregation. It has been observed that the activation of WβC signaling suppresses the BACE1 activity thereby inhibiting amyloid plaque formation [60,61] and the loss of WβC signaling induces AD-like neuropathological abnormalities in mice [60]. The PSEN1 gene regulates hippocampal synaptic plasticity, Ca^2+^ homeostasis, and Aβ production, thereby maintaining brain homeostasis. Further, mutations in the PSEN1 gene fail to regulate these functions leading early onset of familial AD (FAD) [62,63,64]. Recent studies uncover that PSEN1 and its mutation are considered to negatively regulate the WβC signaling by promoting GSK-3β phosphorylation [65], and PSEN1 mutations are reported in AD patients, with enhanced degradation of β-catenin [66]. Cumulatively, PSEN1 mutations negatively regulate WβC signaling, thereby causing AD. Astrocytes release WNT 7a (anti-aggregate tau mutant) and activate WβC signaling, thereby enhancing the neurogenesis in AD [67,68]. 

Age is the major risk factor for the development of AD. The aging brain is found with downregulated WβC pathway [69] with decreased expression of WNT 2-4, WNT 7b, WNT 10b, DVL1, and DVL2 and increased expression of DKK1, which is a WNT antagonist [70], thereby causes an aging-mediated decrease in neurogenesis. It has been reported that dysregulation and malfunction of WNT co-receptor, LRP6 is associated with the development of AD in multiple ways, including inhibition of LRP6 expression by APOE4, decreased LRP6 interaction with APP promotes Aβ production and splicing of LRP6 by single nucleotide polymorphisms (SNPs) [57,71,72]. These observations confirm that activation of LRP6 exhibited WβC signaling activation, thereby reducing the risk of AD in aging conditions. Interestingly, during the amyloidogenesis (formation of Aβ), DKK1 is upregulated by the Aβ peptide shutdown WβC signaling [73]. Further, DKK induces AD by enhancing synapse degeneration and enhancing Aβ production. These findings were further supported by observing the increased colocalization of DKK1 in neurofibrillary tangles and dystrophic neurites and hyperphosphorylated tau protein in postmortem AD brains [55,73,74]. Further, treatment with DKK1-neutralizing antibodies [75] reduces the risk of AD. These findings emphasize that DKK1 promotes Aβ production and synapse degradation through downregulating WβC signaling. Further research needs to validate the pharmacological inhibition of DKK1 is beneficial to mitigate AD or not.

The blood–brain barrier (BBB) is composed of endothelia cells, pericytes, and astrocytes with several numbers of tight junctions [76]. The BBB protects the brain from external neurotoxic agents such as microbes, cells, and blood-derived harmful substances [77,78]. BBB leakage is considered one of the biomarkers for cognitive impairment in AD and the aging population [79]. Sweeny et al. identified that the compromised BBB enhances its permeability, decreases glucose transport, promotes the accumulation of neurotoxicants, degeneration of endothelial cells and pericytes, and infiltration of inflammatory markers [80], leading to AD and PD [81]. P-glycoprotein (Pgp) regulates the entry of drugs, chemicals, and other substances into the brain. Pgp is essential for the transport and clearance of Aβ around the blood and BBB. It was suggested that Pgp clears the excessive amounts of Aβ and impaired Pgp efflux mechanisms due to BBB breakdown leading to failure in Aβ clearance [82]. WβC activators such as WNT 7a/WNT 7b regulate the BBB formation and integrity and CNS angiogenesis via Reck and Gpr124 proteins [83]. These WNT ligands activate the WβC pathway in endothelial cells (EC) through upregulating tight junction proteins such as claudin 1, 3, and 5, which enables the formation of connections between multiple cells, which results in BBB formation and maintains its integrity [68,84]. These things considered, the activation of WβC signaling enhances the glucose transporter 1 (GLUT1) [85] and Pgp expression in EC cells [86], thereby regulating the BBB formation and integrity. Together, WβC signaling activation is required to regulate the tight junctions, GLUT1, and Pgp proteins to maintain the BBB integrity and function. However, limited information is available on BBB regulation by WβC signaling in AD. Therefore, in-depth knowledge about the crosstalk between WβC signaling and BBB regulation in AD has to be further investigated.

There is mounting evidence that the development of Alzheimer’s disease interacts extensively with immunological processes in the brain and extends beyond the neuronal compartment [87]. The interaction of pattern recognition receptors in glial cells and astrocytes triggers the innate immune response cells to release inflammatory mediators, which further extent and progress AD [87,88]. Microglia highly express the triggering receptors expressed on myeloid cells (TREM2), which is the major culprit in developing neuroinflammation in AD [89]. Surprisingly, WNT activators WNT3a, TDZD-8, and LiCl treatment activate the TREM2-mediated glial cell survival through WβC activation [90]. Moreover, postnatal deletion of LRP6 activates microglia and neuroinflammation in AD [57]. These findings imply that deletion of LRP6 (WNT activator) activates the inflammation; on the contrary, other WNT ligands promote glial cell survival through activation of WNT signaling. Hence, there is a dilemma about WβC signaling and neuroinflammation in AD. Therefore, these conflicting results pertaining to WβC signaling in microglia activation and neuroinflammation has to be further explored in AD [91].

Prevention of β-catenin phosphorylation and degradation is most important for the activation of WβC signaling. GSK-3β is known to phosphorylate the β-catenin and promote its degradation [92]. Upon WNT, protein binding to Fzd/LRP can inhibit the GSK-3β, thereby activating WβC signaling [7]. AD patients were diagnosed with enhanced GSK3β activity [93]. In addition, GSK-3β also promotes tau hyperphosphorylation of TauAβ deposition, and microglia-induced neuroinflammation, which eventually leads to memory loss in AD patients [92,94]. Therefore, GSK-3β inhibition promotes the activation of WβC signaling and inhibits the above-mentioned pathological hallmarks. There are several GSK-3β inhibitors have been found to halt AD-mediated pathological events such as tau phosphorylation, Aβ formation, and cognitive and memory impairment [95,96,97], suggesting that GSK-3β inhibitors are the future molecules to treat AD. However, many more studies need to be executed to validate GSK-3β as a potential target for the management of AD.

Given the importance of the WβC signaling role in the pathogenesis of AD, several attempts were made to activate or stabilize this pathway to reduce the Aβ production and Aβ aggregation. Apart from WNT proteins, it was proven that long-term moderate exercise and environment enrichment could activate WβC signaling by increasing LRP6 and WNT3a protein and decreasing DKK1 protein expression [98,99]. Comparatively, elderly women have more prevalence of AD than younger women because of decreased estrogen levels [100]. It has been found that long-term suppression of estrogen in rats is associated with elevation of basal DKK1 expression leading to suppression of WβC signal in the hippocampus. When estrogen levels were elevated, it inhibited DKK1 and thereby attenuated tau phosphorylation and elevated WβC signaling. These findings emphasize that decreased estrogen in older women promotes the DKK1 (antagonist of WβC signal) thereby develops AD. Therefore, DKK1 suppression is considered a potential therapeutic target for the management of AD [49,101]. It was further supported by DKK1 inhibitors such as antisense oligonucleotides (ASO) and IIIC3 (gallocyanine) that attenuated the neuronal apoptosis, and tau phosphorylation implies that DKK1 inhibition is a promising therapeutic target for the management of AD [102,103]. However, further investigations are required to identify the potential pharmacological agents which can inhibit the DKK1. WNT activators like, WASP-1 [104], curcumin [105], simvastatin, and lovastatin, improves the memory and cognitive function AD [106,107]. Another important protein NeuroD1 (which is responsible for the differentiation of induced pluripotent stem cells into neural progenitor cells) and survivin, an inhibitor of neuronal apoptosis are activated by WβC signaling activators leading to neurogenesis and attenuation of neurodegeneration in AD [108,109].

**Figure 2:**
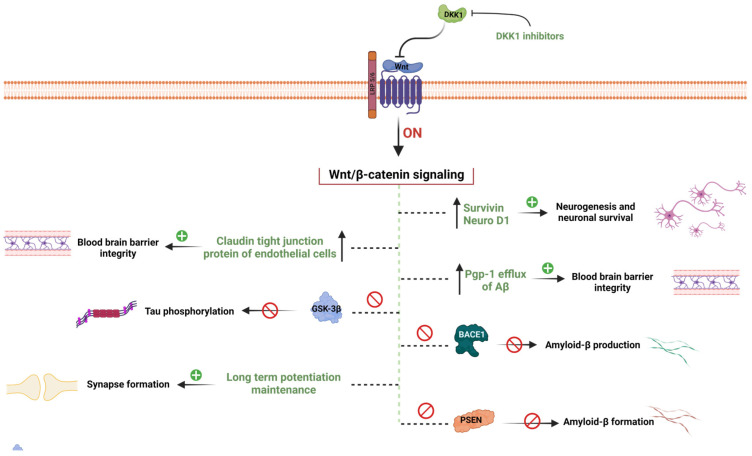
WβC signaling mediated hypothesis of AD. WβC signaling inactivation leads to the pathogenesis of AD. Inactivation of WβC decreases the survivin, and neuroD1 enhances the BBB permeability by decreasing tight junction proteins, enhances Aβ production by promoting BACE1 and PSEN1 mutations, decreases Pgp-1 efflux of Aβ elimination form the brain, decreases the synapse formation, enhances the GSK-3β mediated tau phosphorylation, and promotes DKK1 levels. Pharmacological activation of WβC signaling reverses these pathological abnormalities in AD.

Based on the above literature, several endogenous WNT proteins such as WNT 3a, WNT 7a/7b, WNT1a, and LRP6 activate the WβC signaling, whereas WNT antagonist GSK-3β and DKK1 inhibit the WβC signaling in AD. Comprehensively, WβC signaling promotes neurogenesis and prevents neuronal loss in AD in multiple ways, including inhibition of tau phosphorylation, Aβ production, DKK1 suppression, GSK3β inhibition, APP suppression, BBB integrity enhancement, BACE1 inhibition, increased expression of NeuroD1 and surviving, and neuroinflammation inhibition, which eventually enhances synaptic plasticity in animal models as well as in AD patients [28] (Figure 2). Though the WNT activators showed promising results in preclinical studies, their clinical validity has to be investigated. Therefore, clinical trials in order to prove the WβC signaling as a potential target to combat AD has to be investigated.

## 3. WβC Signaling in Parkinson’s Disease

Parkinson’s disease (PD) is one of the most frequently occurring neurodegenerative diseases after AD [110,111,112]. PD is characterized by the progressive degeneration of dopaminergic neurons in the substantia nigra pars compacta of the brain. This degeneration reduced the levels of dopamine and accumulation of excessive α-synuclein, leading to the formation of Lewy bodies [111,113,114,115,116]. Dopamine (DA) is the major neurotransmitter that regulates motor activity [117,118,119,120]. Due to the degeneration of DA neurons, motor activity is uncontrolled by DA leading to the occurrence of tremors, rigidity, bradykinesia, and impairments in postural stabilities [121,122,123,124]. Several research studies indicate that PD patients diagnosed with these motor impairments are also found with reduced DA levels, increased accumulation of α-synuclein, and Lewy bodies formations which are considered diagnostic hall markers of PD [125]. Approximately 95% of PD cases are sporadic, and about 5% are non-sporadic [126]. As of now, anti-PD therapy relies on dopamine replacements and provides symptomatic relief. Indeed, PD disease-modifying therapy is not available. However, several attempts were made to identify a disease-modifying agent in PD by targeting the inflammation, leucine-rich repeat kinase 2 (LRRK2), DJ-1, parkin, PTEN-induced kinase 1 (PINK1), α-synuclein, and vps35 roles in the pathogenesis of PD. However, none of these proven to be disease modifying targets in PD and more research need to be conducted to validate these targets [127,128].

Apart from motor symptoms, PD also exhibits several non-motor features like hyposmia, sleep disturbances, cognitive decline, and other mental health disorders [129]. PD is most often considered an age-related neurodegenerative disease [130]. In PD, neurogenesis is diminished because of multipotent undifferentiated and self-renewing precursor cells [131]. The hippocampal dentate gyrus contains subventricular zones (SVZ) and subgranular zones (SGZ) that produce new neurons which recognize odor, spatial learning, and contextual memory capabilities [132]. It has been reported that early degradation of SVZ and SGZ is reported to be involved in premotor symptoms in PD. Therefore, a disease-modifying target with an enhancement of neurogenesis could be a potential target to treat PD [133]. Episcopo et al. and others extensively studied the role of WβC signaling in the pathogenesis of PD. This research group observed that the WβC signaling enhances neurogenesis by regulating SVZ and SGZ, thereby regulating adult dopaminergic precursors and maintaining the homeostasis of neural stem cells (NSC) proliferation, differentiation, and integration [134,135]. Additionally, the WNT pathway regulates mitochondria dynamics, apoptosis, and cell cycle in NSC [136,137]. Astrocytes and microglia cells are thought to play a crucial role in the age-dependent progression of PD by releasing oxidative stress and inflammation mediators [138]. In fact, age-dependent inflammation and oxidative stress downregulate WβC signaling. Bianca et al. proposed that there is a synergy between the upregulation of proinflammatory mediators, downregulation of antioxidants, and decline in astrocyte-mediated WNT activation cause impairment in NSC neurogenesis in PD [1]. Lithium, a potent activator of WβC signaling that reportedly ameliorates astrocyte-mediated oxidative stress and inflammation, thereby halts the progress of PD and enhances the neurogenesis [5]. Currently, Lithium has shown promising results in preclinical studies. However, its clinical validity has to be further addressed. Therefore, inhibition of inflammation, oxidative stress, and inactivation of astrocytes by WβC signaling activates neurogenesis in PD.

The physiological or pathological events like cell proliferation and survival are regulated by WβC signaling whereas WNT/Ca^2+^ and WNT/PCP regulated cell differentiation, cell polarity and migration [6]. Among these, WβC and WNT/PCP play a vital role in SVZ and SGZ-mediated neurogenesis [139]. Further, the activation or inactivation of WNT signaling depends on the dynamic interplay between endogenous WNT agonists and antagonists, which strengthen or weaken WβC signaling [7]. Canonical WNT ligands include WNT1-3a, WNT8, and WNT8a, and non-canonical WNTs are WNT4-7a and Wnt11 are involved in the activation of WβC signaling [140]. WβC signaling is involved in regulating the genes that cause dopaminergic progenitors [141]. For example, GBA1 mutations were reported to downregulate the WβC signaling in human induced pluripotent cells (IPSCS) to differentiate into dopaminergic neurons. WNT activator CHIR99021 facilitated the differentiation of IPSCS into DA neurons [142]. GSK-3β, CK1-α, axin-1, and APC regulate the β-catenin, SVZ, and SGZ, thereby controlling neurogenesis [143]. GSk-3β inhibitors, such as indirubin-3-monoxime and kenpaullone, degrade β-catenin, impeding cell proliferation and cell differentiation [144]. Currently, several GSK-3β inhibitors, such as AR-A014418, SC001, Alsterpaullone, TDZD-8, indirubin-30 -monoxime, SB-216763, and TWS119, exhibited antiPD effects by activating WβC signaling in preclinical studies [145]. AZD1080—a GSK-3β inhibitor—is currently under clinical trials for the management of PD. Overall, inhibition of GSK-3β enhances the β-catenin stabilization, WβC signal activation, NSC proliferation and neurogenesis in SGZ and SVZ road to use GSK-3β inhibitors to enhance stem/precursor cell therapy in PD [134,144].

Short Noncoding RNAs are critical regulators of WNT signaling and vice versa; WNT signaling components can modulate miRNA activity [146]. MIR-135A2 and LIM homeobox transcription factor 1-beta (LMX1B) modulate WNT1/WNT signaling, thereby maintaining the midbrain DAergic progenitor pool [147,148] through regulating and dicer gene. Dicer is an endoribonuclease essential for miRNA biogenesis and other RNAS-related processes. For the maintenance of adult dopaminergic neurons, a reduction of Dicer in the ventral midbrain (VM) and altered miRNA expression profiles has been observed in laser-microdissected dopaminergic neurons of aged mice [149]. MIR-21 also promotes NSC proliferation and neural differentiation via the WβC signaling pathway [150]. Therefore, the above findings revealed that the crosstalk between WβC signaling and the dicer gene is important for the determination of the fate of neurons through the regulation of miRNAs. Currently, limited data is available on the role of miRNA-mediated WNT pathway regulation in PD. Further studies are required to retrieve the therapeutic efficacy of miRNA-based treatment against PD.

A wide range of chemicals has been used to modulate the WβC pathway, including anti-inflammatory agents, antioxidants, antibiotics, herbal derivatives, neurotrophic factors, exercise optical depolarization electromagnetic fields, neural agonists, and nanoparticles. Antioxidants such as curcumin, apomycin, and acetyl l carnitine, mitigated oxidative stress by activating the WβC pathway. Minocycline, curcumin, and chemokines (ccl3, cxcl10, and cxcl11) upregulated the WβC pathway through the regulation of inflammation. Optogenetic activation enhances DA neuron differentiation of NSC by activating the WβC pathway. Amiodiquine, a neural agonist, enhances hippocampal neurogenesis via WβC signaling [1]. Therefore, these chemical and pharmacological activations of WβC signaling could be beneficial in the management of PD. However, further studies are required to clarify the disease-modifying capabilities of WβC signaling activation in PD.

**Figure 3:**
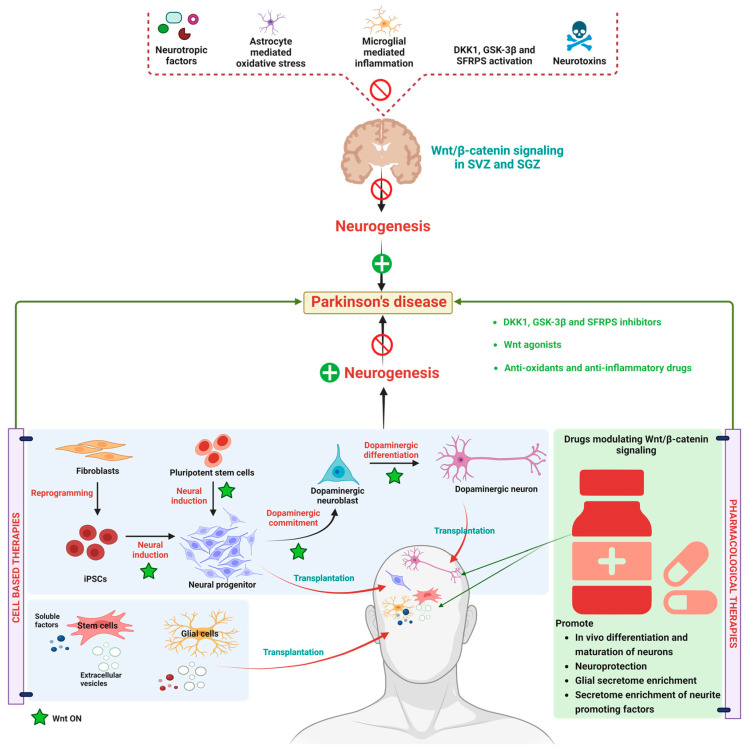
Alteration in WβC signaling in Parkinson’s disease. WβC signaling is downregulated in PD due to a reduction in neurotrophic factors, astrocyte-mediated oxidative stress, microglial-mediated inflammation, WβC antagonism by GSK-3β, DKK1, SFRPS, and neurotoxins eventually inactivated the WβC signaling in SVZ and SGZ resulting in neurodegeneration. Stem cell-based therapies induce the WβC activation, thereby promoting dopaminergic neurogenesis in PD. Pharmacological activation of WβC signaling by inhibiting GSK-3β, DKK1, SFRPS, and antioxidants. Anti-inflammatory therapies reverse the neurodegeneration in PD.

Currently, drugs like L-Dopa, alone or in combination with catechol-O-methyltransferase (COMT) inhibitors/monoamine oxidase B (MAO-B) inhibitors, are used to relieve the symptomatic problems associated with PD. These therapeutic agents cannot prevent/regenerate the progressive loss of DA neurons [151,152]. Therefore, these agents are primarily considered in PD treatment to reduce motor problems in PD. It has been reported that with the progression of PD, the responsiveness of these agents declined, and the longer duration of these medications produces side effects like dyskinesia and cheese reaction, etc. [153]. Moreover, 5% of PD cases are familial forms, which has been occurred due to mutations in LRRk2, VP535, PINKI, α-synuclein, DJ-1, and parkin genes, where as 95% of cases of PD have occurred due to lifestyle factors [127]. In view of the current above-mentioned treated drugs and their ineffectiveness with disease progression, there is a need for disease-modifying strategies to treat PD. Therefore, research has combined neuroprotective agents and neurogenerative agents to combat PD [154,155]. As per current knowledge about PD treatment, neuroprotective stem cell-based therapies seem to be more fruitful. Recent findings from in vitro and in vivo studies revealed that stem cell secretion by a combination of neurotrophic and immune factors and vesicular fractions of neural and non-neural stem cells could be beneficial for the treatment of PD [156]. WβC (canonical) pathway in not only involved in embryonic development but also preserves adult tissue homeostasis [157]. Owing to multiple functions of WβC functions, such as neuronal differentiation, synapse formation, and neurogenesis, WβC dysregulation is considered the new pathological factor which causes PD [1]. The balance between WNT on and WNT off signaling in healthy midbrain DA neurons are regulated by microglia and astrocytes. Further, the cross-talk between astrocytes and microglia is important to combat midbrain injury through secreting WNT ligands and expressing WNT-frizzled receptors and secreted frizzled-related proteins (sFRPs) [158]. The imbalance between this cross-talk promotes neuroinflammation and decreases neuroprotection which eventually leads to neuron loss [27]. Therefore, pharmacological activation of the WβC pathway could be a promising target to combat PD. The data from preclinical and clinical studies produced promising results in combating PD with the use of WNT1, WNT2, WNT3, WNT31, WNT 8, and WNT8a agonists and GSK-3β inhibitors and DKK1 inhibitors. Natural compounds like curcumin, resveratrol, ginosides, and salidoside activated the WβC pathway and exhibited neurorestorative properties in PD [155]. Neurotropic factors, such as brain natriuretic peptide (BNP), atrial natriuretic peptide (ANP), and c-type natriuretic peptide (CNP), are involved in neural development differentiation [156]. These neurotrophic factors are reported to improve the dopaminergic neuron differentiation and maturation of hiPSCs neuronal progenitors by simultaneously activating a WβC signal through modulating frizzled receptors. The modulation WβC signaling pathway is considered a double-edged sword. For instance, irreversible activation of WNT signaling may be protective against neurological disease. In contrast, WβC antagonists are beneficial in cancer treatment which may cause neuronal complications. Therefore, reversible agonists for WβC could be developed and tested for their potency. The detailed molecular mechanism and pharmacological treatment targets are represented in Figure 3.

## 4. WβC Signaling in Huntington’s Disease

Huntington’s disease (HD) is an inherited, incurable, progressive neurodegenerative disorder that is characterized by psychiatric, cognitive, and motor problems [159,160]. The mutations on the Huntington gene on chromosome 4 are mainly responsible for the development of HD [161]. Unlike other neurodegenerative diseases, HD patients were observed with severe neurodegeneration in the thalamus, amygdala, insula, Globus Palladius, and striatum producing multiple symptoms [162]. HD is less prevalent than other neurodegenerative diseases. Currently, 2.7 people out of 1,00,000 are affected by HD with multiple motor symptoms, including problems in speech, gait, and swallowing in the initial stages. Whereas rigidity, bradykinesia, dependence on others, and immobility are observed in the late stages of HD [163]. Cognitive impairment is commonly associated with HD [164]. Initially, cognitive impairments are seen in subcortical regions, whereas in later stages, cognitive decline arises due to cortical and subcortical atrophy, leading to global cognitive dysfunction [165]. HD exhibits a variety of psychiatric symptoms, such as apathy, irritability, and psychosis. Moreover, the severity of motor, cognitive, and psychiatric symptoms vary from person to person because of genetic variations [166]. Around 13% of HD patients exhibit aggression, 7% exhibit psychosis, and 13% have sleep disturbances [167]. To date, tetrabenazine, and deuterobenzene, which are vesicular monoamine transporter 2 (VMAT2) inhibitors, are licensed for the management of HD [168,169]. Olanzapine or sulpiride, which are antipsychotics, are commonly prescribed to improve sleep, mood stabilizer, and increase weight gain [170]. Drugs such as sodium phenylbutyrate, cysteamine, citalopram, memantine, and bupropion are being investigated in clinical trials to combat HD [162]. Additionally, deep brain stimulation (DBS) has improved the chorea in HD. However, further investigations are required to retrieve the therapeutic roles of DBS [171]. However, the existing therapy provides symptomatic relief. Therefore, HD-modifying therapies are sought.

HD is associated with decreased β-catenin levels, and a reduction in TCF-mediated transcription in HD in huntingtin knock-in STHdh^Q111/Q111^ striatal cells has been reported [23]. In another way, GSK-3β phosphorylation inhibited the WβC signaling in HD [172]. Intriguingly, posttranscriptional changes, not GSK-β3-mediated proteasomal degradation, are the cause of the lower levels of β-catenin in HD. Moreover, microRNA-124 (miR-124), which controls β-catenin levels, was found to be 10 folds higher in HD [172,173]. Further, Godin et al. transfected MDCK and HEK293 cells with htt-480-17Q, thereby increasing the β-catenin levels significantly. They found that β-catenin is in phosphorylated form, but this accumulation does not provide the transcriptional activation of WβC signaling. Therefore, these studies revealed [173] that β-catenin levels varied in HD according to in vivo models such as Hdh^Q111/Q111^ and Hdh^Q111/Q111^ knock-in HD mice, drosophila, and post-mortem samples of HD patients [174]. Further, HD neuronal cells derived from iPSCs exhibited significant destruction of the β-catenin complex and increased transcription factor 3 (TF3), frizzled transcripts expression, and enhanced CCND1—a WNT transcriptional target [175]. Moreover, WNT ligands (WNT3, 4,6, 7B, 10A), effectors (TCF3 and TCF4), and downstream targets (AXIN2 and APDCC1) are upregulated on iPSCs-derived microvascular cells from HD patients and WNT inhibition prevented the angiogenic deficits and BBB impairment in HD models [176]. Further treatment with WNT inhibitors such as ICG-001, XAV-339, and indomethacin are reported to have beneficial effects in the management of HD. Unlike other neurodegenerative diseases, in HD, inhibition of WβC signaling is beneficial for the treatment of HD. At present, in vitro and in vivo inhibitors of WβC signaling are reported to mitigate the HD. However, whether the pharmacological inhibition of WβC signaling is beneficial in HD patients yet to be identified. Therefore, future studies can target this pathway as a potential target to attenuate HD.

## 5. WβC Signaling in Amyotrophic Lateral Sclerosis

Amyotrophic lateral sclerosis (ALS), also termed Lou Gehrig disease, is characterized by the degeneration of motor neurons. ALS etiology is not proven [177]. Like other neurodegenerative diseases, multiple pathways are involved in the pathogenesis of ALS. It has been observed that around 5–10% of cases of ALS are familial, whereas 90–95% are sporadic [178]. In the last three decades, at least 25 mutant genes have been identified to be involved in ALS pathology, which is involved in protein homeostasis, RNA binding proteins, and cytoskeletal proteins, which contribute to inflammation, glutamate accumulation, and calcium imbalance [179,180,181]. The ALS cases are escalating day by day, and ALS is becoming a global burden. ALS is characterized by lower and upper motor neuron degeneration in the motor cortex, spinal cord, and brain stem; due to lower and upper motor degeneration eye, sphincters, and respiratory neurons go out of control, and eventually, death occurs [182,183]. As the symptoms of ALS are similar to other motor neurons, it is difficult to diagnose ALS in the early stages. However, recent studies suggest that ALS can be diagnosed by combining clinical, electrophysiological, neuropathological, and neuroimaging studies to provide a robust diagnosis of ALS [184,185,186,187].

The majority of ALS patients have lower and upper motor function failures. Therefore, during the management of ALS, occupational therapy, physical therapy, respiratory therapy, social work, nursing, and dietary therapy can be provided to ALS patients [188]. Drugs like mexiletine are used to treat painful spasms [189]. Levetiracetam, baclofen, gabapentin, and tizanidine are used to control motor problems in ALS [190]. Botulinum toxin (muscle spasms), atropine, amitriptyline, hyoscyamine, and glycopyrrolate are used for the management of Sialorrhea in ALS [191,192]. Pharmacological agents, such as dextromethorphan-quinidine [193], riluzole [194], and edaravone [195], are shown to be effective in ALS. However, most of these drugs are used for symptomatic relief. Therefore, disease-modifying agents need to be identified.

WβC signaling maintains neuronal differentiation, neurogenesis, synaptic stability, and plasticity [196]. In view of these physiological roles, WβC is chosen as a promising target for the treatment of neurodegeneration diseases. In diseases such as AD and PD, WβC signaling is down-regulated, whereas the mRNA and protein levels of WNT agonists, receptors, co-receptors, and modulators are found to be altered in ALS transgenic mice [197,198] and ALS postmortem spinal cord issues [199]. SOD1-G93A transgenic mice are widely used to study the pathology of ALS. These mice had an elevated expression of WNT proteins such as WNT1,2,3A, and 7A [200], which are considered WβC activators [201,202]. GSK-3β is considered as downregulates WβC by degrading. β-catenin in the cytoplasm resulting in decreased β-catenin levels at the nucleus. GSK-3β levels are upregulated in the spinal cords [203] and brains [204] of ALS patients and animals. Further, GSK-3β also phosphorylates the TAR DNA binding protein 43 (TDP-43), which is a potent inflammatory mediator [205]. Thus, these findings emphasize that GSK-3β degrades β-catenin and simultaneously promotes neuroinflammation, thereby causing ALS. Currently, Tideglusib—an inhibitor of TDP-43—is under phase 2 clinical investigation (NCT05105958) to treat ALS [206]. In contrast, β-catenin levels in the ALS motor neuron levels are significantly elevated [202,207]. These findings conclude that WβC signaling is hyperactivated in ALS transgenic mice. However, further investigations are required to determine if the hyperactivation of WβC is beneficial or harmful in ALS.

WNT/Ca^+^ and WNT/PCP pathways are β-catenin-independent signaling pathways that regulate calcium levels by regulating Ca^2+^ release for the endoplasmic reticulum [7]. The binding of WNT ligands to FZD receptors activates the Dsh, thereby activating phospholipase C (PLC), diacyl glycerol (DAG), and inositol triphosphate 3 (IP3), CAMKII, calcineurin NFAT pathways—eventually leading to Ca^2+^ release [7,12]. Further, Ca^2+^ controls the cell fate and migration in the β-catenin independent pathway [7]. It has been reported that the number of FZD2-positive astrocytes is substantially increased in ALS mice and patients [25,208], thereby enhancing the inflammatory responses in ALS, leading to neurodegeneration [209].

Astrocytes, microglia, and oligodendrocytes maintain homeostasis in the brain and are reported to contribute to the pathogenesis of ALS [177]. Jiang et al. reported that motor neurons, astrocytes, microglia, and oligodendrocytes express high levels of WβC signaling and β-catenin activation in ALS [177]. Precisely, alterations in these events cause ALS in multiple ways. Firstly, high levels of β-catenin are accumulated in the cytoplasm of motor neurons [207]. Secondly, WβC activation leads to astrogliosis in astrocytes [182]. Thirdly, the activation of WβC leads to the transformation of microglial cells into proinflammatory microglial cells [210]. Fourthly, oligodendrocytes play a diverse role in the pathogenesis of ALS. In regenerated oligodendrocytes, WβC activation leads to delayed maturation, impairment in myelin repair efficiency, formation of immature myeline sheaths, and inflamed myelin sheaths, eventually leading to non-myelinating oligodendrocytes [211,212]. Whereas, existing oligodendrocytes restore the myelin structure and oligodendrogenesis [177]. Oligodendrocyte development during physiological and pathological conditions is driven by WβC signaling [213]. Indeed, WNT3A-mediated activation of WβC signaling and inhibition of GSK-3β enhances oligodendrocyte proliferation and restores the myelin [213,214]. Therefore, these findings conclude that WβC signaling activation by GSK-3β inhibition has positive effects on existing oligodendrocytes. However, activation of WβC signaling in regenerating oligodendrocytes produces the detrimental effects. However, the reasons for the elevation of WβC signaling in regenerating oligodendrocytes and decreasing WβC signaling in existing oligodendrocytes is unknown. Therefore, future studies are required to reveal the possible mechanisms of these changes in WβC signaling upregulation and downregulation in oligodendrocytes.

The neuromuscular junction (NMJ) is the junction between skeletal muscle and spinal cord motor neurons that regulates motor function. Any disturbance in the NMJ structure and function is reflected in neuromotor behavioral abnormalities [215]. In ALS, NMJ loses its connection—leading to paralysis or neuromuscular problems in the early stages of ALS [216,217]. The acetylcholine (Ach) and acetylcholine receptors (AchR) maintain the integrity and neuronal communication at NMJ and connect the neuronal responses to muscles [218]. Therefore, any perturbations to these Ach and AchR can directly impact motor performances. AchR in the synaptic site forms the NMJ [219]. LRP4 is an essential protein for postsynaptic differentiation in the early stages of synapse formation [220]. Motor nerve terminals release agrin, which then combines with LRP4, thereby activating the muscle-specific kinase (MUSK). The formation of the AGR-LRP4-MUSK complex stabilizes postsynaptic differentiation [221,222]. LRP4 is known to inhibit the LRP6-mediated activation of WβC signaling [223]. Therefore, these findings indicate that LRP4 is essential for NMJ formation. WNT3a is reported to inhibit the agrin-mediated AchR clustering at NMJ, thereby impairing motor function [224]. However, WNT4 and 11 enhance AchR clustering and motor axon outgrowth, activate WβC, and enhance mRNA levels of AchR [222] in healthy conditions. WNT11, or decreased WβC inactivation, significantly decreases clustered AchR and motor nerve terminal branching. NMJ in mammalians is formed by WβC and WNT/PCP signaling mediated by WNT4 and WNT11 [222]. Therefore, these results indicate that the WNT agonists have a diverse role in the formation of NMJ. However, further studies are required to reveal the WNT ligands and their role in the formation or destruction of NMJ. Skeletal muscles and NMJ are the earliest and one of the primary sites that are damaged in ALS. Therefore, identifying the NMJ and skeletal muscle abnormalities in early stages prevents further motor problems in ALS by starting early interventions in ALS patients [225]. Frizzled-related protein (FRZB)—a WNT antagonist identification is considered the molecular sign of muscle denervation in ALS [226]. Furthermore, recent studies explored that an increase in MUSK may be beneficial in ALS patients [227] and in SOD1-G93A mice is suffice to produce sustained NMJ synapses and improve muscle function [228]. Further, these findings supported by administering a MUSK agonist antibody preserved the NMJ innervation in ALS [229]. Furthermore, LRP4 autoantibodies are found in the late stages of ALS patients’ serum and cerebrospinal fluid (CSF). Therefore, LRP4 antibodies appeared in worsened conditions of ALS, and treatment with LRP4 anti-autoantibodies may be beneficial for late-stage ALS patients to develop NMJ [230]. Hence this literature confirms that alterations activation of WNT/PCP by WNT4 and WNT11, presence of FRZB, activation of MUSK, and LRP4 autoantibodies are used to diagnosis for the early and late stages of ALS, thereby early interventions can be initiated.

**Figure 4:**
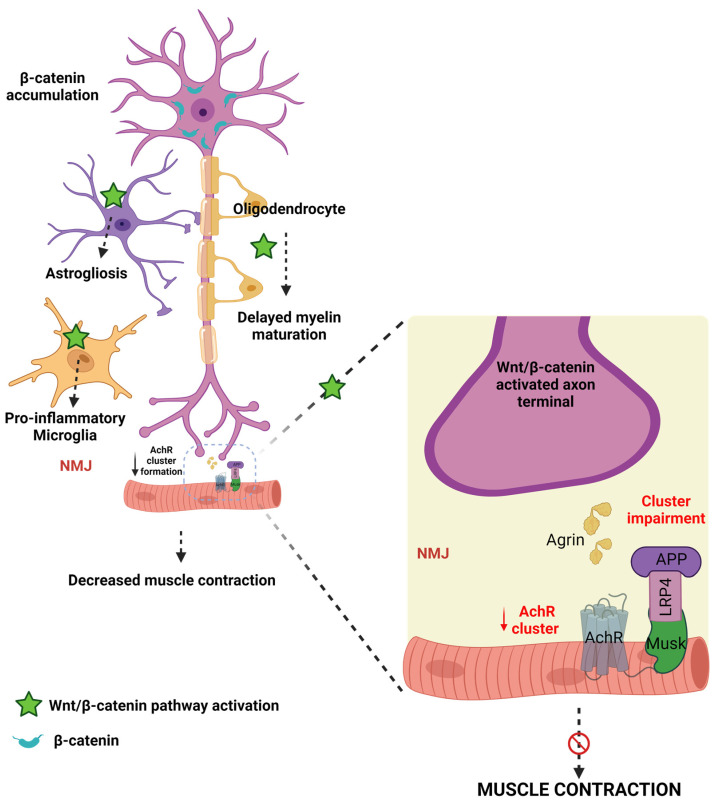
The connection between WβC signaling and ALS. WβC signaling is upregulated in ALS. Microglia, astrocytes, and oligodendrocytes promote demyelination and inflammation and impair the neuromuscular junction in motor neurons. AchR clustering formation is important for NMJ formation. The agrin-LRP-MUSK complex is essential for the formation of NMJ. Upregulation of WβC signaling impairs the formation of Agrin-LRP4-MUSK, resulting in impaired AchR clustering in the NMJ.

Based on this evidence, WNTS and WNT receptors involved in the canonical- and non-canonically mediated WβC signaling have contributed to the dysfunction of the overall motor unit from axonal terminal denervation to the death of motor neurons. Moreover, WβC activation causes the denervation of neurons, oligodendrocytes, astrocytes, and microglial dysfunction (Figure 4). Therefore, WβC inhibition, MUSK agonists, and LRP4 activation may be beneficial for the management of ALS. Further, the crosstalk between WβC and ALS progression has to be investigated.

## 6. WβC Signaling in Multiple Sclerosis

Multiple sclerosis (MS) is a chronic, severe, inflammatory, and autoimmune disease distinguished by demyelination of white matter of the central nervous system. In particular, persistent inflammation causes axonal myelin sheath destruction in the brain and spinal cord, which can lead to neuronal death and the progressive loss of neurological functioning [231]. Microglia and Th17 cells produce the proinflammatory mediators that are responsible for the immunogenic reaction on the myelin sheath, thereby causing demyelination [232]. TCF-1, a Wnt transcription factor, acts as an epigenetically negative regulator of Th17 differentiation of thymus and peripheral T cells. Deletion of TCF-1 has been linked to enhanced interleukin-17 (IL-17) gene expression and enhanced Th17 differentiation in experimental autoimmune encephalomyelitis [233,234]. Similarly, in dendritic cells generated from experimental autoimmune encephalomyelitis (EAE), animals exhibited loss of LRP5, LRP6, or β-catenin resulting in increased proinflammatory cytokine production and increased Th1 and Th17 cell polarisation, thereby increasing the EAE severity [235]. LiCl pre-treatment, which is a GSK-3β inhibitor in EAE animals, reduced leukocyte infiltration, demyelination, and microglia activation in the mouse spinal cord. In fact, this inhibition of GSK-3β activity is associated with a reduction in nuclear factor-kappa B (NF-Kb) activation [236]. In contrast to these findings, upregulation of WNT3a and β-catenin levels are significantly elevated in pain centers in the spinal cord (dorsal horn). Similarly, WNT5a (noncanonical) is overexpressed in spinal cords. The pharmacological inhibition of β-catenin by indomethacin and WNT5a by Box5 significantly ameliorated the allodynia and hyperalgesia in EAE-induced pain in experimental animals [237]. Surprisingly, downregulation of WβC signaling promotes myelination, and upregulation of WβC signaling inhibits remyelination in MS could be due to the time-dependent progression of MS [238,239]. Therefore, further studies are required to disclose the precise role of WβC signaling in the pathogenesis of MS. Breakdown of BBB is considered an early pathological marker for the diagnosis of MS. Dysregulation of BBB enhances the permeability and neutrophil infiltration in MS [240]. WβC signaling maintains the BBB integrity and function by regulating endothelial cells and tight junction proteins [241]. Recent findings indicated that WβC signaling is upregulated in CNS endothelial cells, thereby enhancing the endothelial cell breakdown, infiltration of CD^+4^ positive T cells, and demyelination. Further, genetic suppression of WβC signaling before initiation of the onset of disease aggravates these pathological events. Inhibition of WβC signaling does not prevent the progression of MS. Therefore, reactivation of WβC signaling partially restores the endothelial function and inhibits infiltration in MS [242]. However, more studies are required to explore the BBB role in the pathogenesis of MS.

## 7. WβC Signaling in Spinal Muscular Atrophy

Spinal muscular atrophy (SMA) is an inherited neurodegenerative disease that mainly destroys the alpha motor cells in the horn cells of the spinal cords [243]. Homozygous deletions of survival motor neurons 1 (SMN1) on chromosome 5q13 are responsible for 95% of SMA, called as SMN dependent SMA, whereas the rest of the 5% SMAs are called SMN-independent SMA [244]. SMA causes death within weeks of birth or produces significant motor abnormalities during adulthood. Approximately one in every 6000–11,000 humans is affected by SMA [245,246]. SMN protein works distinctly at neuronal cytoplasm and neuronal nuclear activity [245]. In the case of neuronal cytoplasm, SMN is pivotal for mRNA transport though axons, regulation of actin dynamics, and synaptic vesicle release [247]. Whereas nuclear SMN forms small nuclear (snRNA), which then plays a crucial role in the formation of splicesome, which is responsible for the removal of introns in pre-mRNA into functional mRNA [247]. Due to the deletions of SMN, cytosolic and nuclear functions are altered, and failure to splice leads to the dysfunction of protein synthesis in neurons [248]. SMN1 deletions in motor neurons throughout the body are impaired, and signs and symptoms vary as the neuronal motor impairments are region-specific. For example, breathing difficulties, respiratory failure, respiratory infections in the lungs, dysphagia in the GI tract, cardiomyopathy (heart), spasticity, muscle weakness, fatigue, hip dislocation, scoliosis, kyphosis, and contractures are seen in skeletal muscles. Because of these symptoms, there is a high rate of mortality in children and severe motor impairments in adults. Pulmonary, gastrointestinal, and orthopedic supportive care [249,250,251], along with drugs like nusinersen [252], onasemnogene abeparvovec [253], risdiplam [254], and botulinum toxin [255], are used for the treatment of SMA.

It has been reported that in SMA, laminin A/C levels are increased and inevitably cause myocardial rigidity, thereby causing cardiomyopathy [256]. SMN-dependent SMA exhibits low levels of ubiquitin-like-modifier activating enzyme 1 (UBA1), which is encoded by the UBE1 gene and is linked to producing the crosstalk with lamin A/C [257,258]. UBA1 activates the ubiquitin-mediated proteasomal degradation of β-catenin [24]. The levels of UBA1 are significantly reduced in SMN-dependent SMA, and pharmacological and genetic suppression of UBA1 contributes to the pathogenesis of SMN-dependent SMA [24]. Further, activation of UBA1 enhances the neuromuscular (motor) function in zebrafish, suggesting that UBA1 is critical for the amelioration of SMA [259]. Recent studies revealed that lamin A/C, UBA1, and β-catenin levels are interlinked with each other in the contribution of the pathology of SMN-dependent SMA. Glycine t-RNA synthetase (GARS) mutations are responsible for the inhibition of UBA1, thereby causing SMA. Further, the increased levels of GARS and LMNA enhance the β-catenin levels, which contributes to the pathogenesis of SMA [259,260]. GARS protein, namely the aminoacyl t-RNA synthetase interacting multifunctional proteins (AIMPS), such as AIMP2, enhance the β-catenin levels reported to enhance the SMA [261]. Wishart et al. reported that pharmacological inhibition of WβC signaling using quercetin reduced the neuromuscular abnormalities in preclinical animal models such as drosophila, mouse, and zebrafish [24]. These observations emphasize that WβC signaling is overactivated, thus contributing to the pathogenesis of SMA.

**Figure 5:**
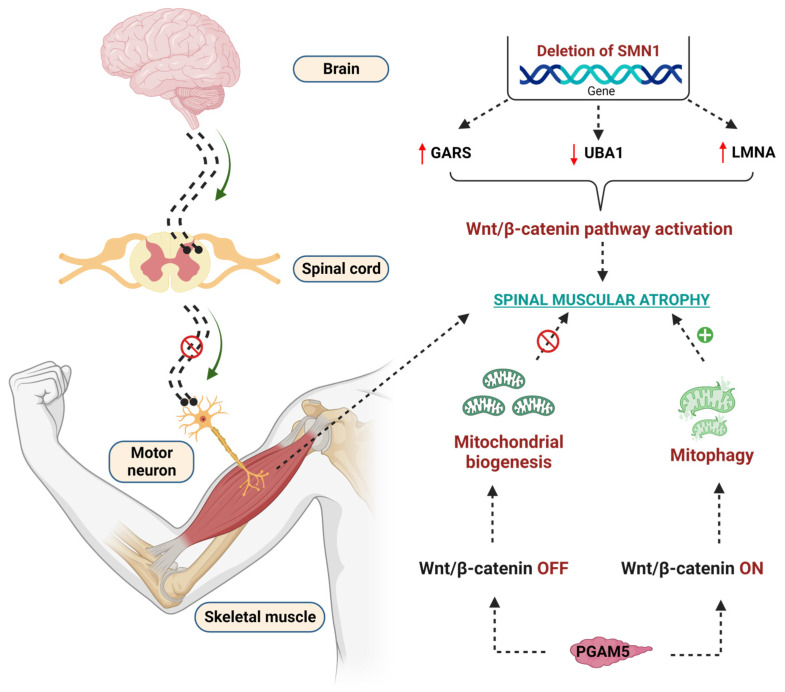
Effect of WβC signaling overactivation in the pathogenesis of SMA. Deletion of the SMN1 gene enhanced the GARS, reduced the UBA1, upregulated the LMNA genes, and overactivated the WβC signaling to lead to SMA. PGAM5 regulates the mitochondrial function in WβC signaling-dependent manner. PGAM5 promotes mitochondrial biogenesis through the WNT-off mechanism. Oppositely, PGAM5 promotes mitophagy through the WNT mechanism.

Mitochondria play a crucial role in the pathogenesis of SMA [262]. Mitochondrial biogenesis and mitophagy are two important events to maintain mitochondrial function. In SMA, these two events altered in β-catenin dependent manner [263]. Phosphoglycerate mutase 5 (PGAM) family member 5 (PGAM 5) acts differently at cytoplasmic and nuclear levels to control mitochondrial function [264]. PGAM5 was identified as novel activation of WβC signaling at cytosolic levels, thereby activating mitochondrial biogenesis [264]; whereas the mitochondrial form of PGAM5 suppresses WβC signaling and leads to mitophagy in SMA [265]. These diverse roles of PGMA5 phosphatase in activating or suppressing WβC signaling are important for controlling mitochondrial number in SMA [265,266]. Indeed, the crosstalk between WβC signaling and mitochondria in the pathogenesis of SMA is not clearly understood. Therefore, further studies are required to perform to reveal the crosstalk between WβC and mitochondria in the pathogenesis of SMA. The detailed WβC signaling mediated pathogenesis of SMA is depicted in Figure 5.

## 8. Summary and Future Perspective

Compelling evidence suggests that WβC signaling is essential for neuronal homeostasis. Particularly, WβC signaling activation and inactivation play a pivotal role in the pathogenesis of neurodegenerative diseases. WβC signaling in the brain is pivotal for neuronal differentiation, migration, and neurogenesis. Preclinical and clinical evidence suggests that WβC signaling is involved in the pathogenesis of AD, PD, HD, SMA, ALS, and MS. Based on the above literature, we understand that WβC signaling is downregulated in AD and PD, whereas WβC signaling is upregulated or downregulated in HD, SMA, ALS, and MS. However, these discrepancies in WβC signaling upregulation and downregulation have to be further investigated. WβC signaling directly or indirectly regulates the microglia, astrocyte, and oligodendrocyte function, thereby being involved in the pathogenesis of neurodegenerative diseases.

In AD, WNT proteins, LRP6 and FZD are downregulated, and WNT antagonists GSK3β and DKK1 are upregulated. These two events are crucial for controlling neurodegeneration and neurorestorative. Pharmacological activation of WβC signaling inhibited tau phosphorylation, Aβ production, DKK1 suppression, GSK3β inhibition, APP suppression enhances BBB integrity, BACE1 inhibition, increase in survivin and neuronal expression, inhibited neuroinflammation, which eventually enhances synaptic plasticity in animal models as well as in AD patients [28]. Therefore, accumulating results indicate that activation of WβC signaling is more fruitful than existing therapies to combat AD. The pathophysiological roles of WβC signaling are extensively studied in preclinical studies. However, very few compounds such as lithium chloride, AZD1080, tideglusib, AR-A014418, AZD2858, TCS2002, isonicotinamides, PF-04802367, isoorientin, L803mts, L807mts, Indirubin-3′-monoxime, C7a, and C7b have been shown promising modulation of WβC signaling in preclinical investigations. At present, Lithium chloride, a WNT activator that inhibits GSK-3 β, is in phase 4 clinical trials (NCT03185208) for combating AD. Therefore, potential molecules from these preclinical investigations can be examined in larger-scale research in the future. In PD, WβC signaling is downregulated, resulting in dopaminergic neuron degeneration leading to motor and cognition impairments. The activation of WβC signaling through pharmacological and stem cell-based therapies ameliorated the α-synuclein, accumulated Lewy bodies, activated mutations that cause PD, and enhanced the neurogenesis of dopaminergic neurons. Preclinically, many agents, such as AR-A014418, SC001, Alsterpaullone, TDZD-8, indirubin-30 -monoxime, SB-216763, and TWS119, have been shown to activate WβC signaling and exhibits antiPD [145]. As of now, AZD1080, which is a GSK-3β inhibitor, is currently under clinical trials for the management of PD. Preclinically, all these chemicals exhibited dopaminergic neuronal proliferation. However, their clinical role is not yet investigated. Therefore, future studies need to investigate the neurogenesis potentials of these WNT activators in clinical studies. This has been preclinically achieved with WβC signaling agonists. Interestingly, WβC signaling is upregulated in neurodegenerative diseases like HD, SMA, ALS, and MS. The reasons for the upregulation of these diseases not fully understand. Therefore, future studies should reveal the cause of alterations in WβC signaling. Several WNT agonists and antagonists were tested preclinically to ameliorate these diseases. However, the availability of WβC signaling modulators in clinics is a long wait. Several WβC signaling modulators are under consideration for the management of neurodegenerative diseases in clinical trials. The modulation WβC signaling pathway is considered a double-edged sword. For instance, irreversible activation of WNT signaling may be protective against neurological disease. Conversely, WβC antagonists are beneficial in cancer treatment which may be because of neuronal complications. Therefore, reversible agonists for WβC could be developed and tested for their potency. Corroborating this evidence, WβC signaling is a promising target for the management of neurodegenerative disease.

## Data Availability

There are no data associated with this manuscript.
